# Phosphate Ion-Modified RuO_2_/Ti_3_C_2_ Composite as a High-Performance Supercapacitor Material

**DOI:** 10.3390/nano9030377

**Published:** 2019-03-05

**Authors:** Jie Zhao, Faqian Liu, Weihua Li

**Affiliations:** 1 Key Laboratory of Marine Environmental Corrosion and Bio-Fouling, Institute of Oceanology, Chinese Academy of Sciences, 7 Nanhai Road, Qingdao 266071, China; m15171002248@163.com; 2 University of Chinese Academy of Science, Beijing 100049, China; 3 Qingdao National Laboratory for Marine Science and Technology, Wenhai Road, Aoshanwei, Jimo, Qingdao 266237, China; 4 School of Chemical Engineering and Technology, Sun Yat-sen University, Zhuhai 519082, China; faqianliu@yahoo.com

**Keywords:** supercapacitors, RuO_2_/Ti_3_C_2_ composite, phosphate ion modification

## Abstract

Pseudocapitor materials, usually metal oxides, are used as active materials in an electrode to achieve high energy density. However, these kinds of materials often suffer from poor conductivity and high cost. Herein, a phosphate ion-modified RuO_2_/Ti_3_C_2_ composite is prepared via a chemical solution synthesis followed by an annealing process. In this composite material, Ti_3_C_2_ layers are introduced to improve the conductivity and the binary material is doped with phosphate ions into to increase the number of active reaction sites. As a result, the phosphate ion-modified RuO_2_/Ti_3_C_2_ delivers a high specific capacitance of 612.72 F g^−1^ at a current density of 2 A g^−1^ in H_2_SO_4_ electrolyte. What is more, the capacitance of the phosphate ion-modified RuO_2_/Ti_3_C_2_ can retain 97.95% (600.14 F g^−1^) of the original value even after 10,000 cycles at a current density of 2 A g^−1^.

## 1. Introduction

With the rapid development of electronic products, supercapacitors have attracted much effort in recent years owing to their merits of a quick charge–discharge process, high power density and long cycle lifetime [1,2,3,4,5]. Supercapacitors can be divided into two categories based on their energy storage mechanisms: electrical double-layer capacitors (EDLCs) based on the electrostatic charge accumulated at the electrode/electrolyte interface and pseudo-capacitors based on the reversible faradaic processes [4,6,7,8,9]. The performance of supercapacitors is largely dependent on the properties of electrode materials [10,11]. Porous carbon materials are the most widely used EDLC-type materials in recent commercial applications because they are cost-efficient and easy to synthesize [12,13,14,15]. However, the limited double-layer capacitance of carbon-based materials cannot meet the growing demands of power systems.

An effective option to increase the capacitance is to introduce a pseudo-capacitive material into the electrodes. Traditional pseudo-capacitance materials include metal oxide and conductive polymer materials [16,17,18,19]. Among the various metal oxide reported so far, ruthenium oxide (RuO_2_) has attracted great attention as an ideal candidate in view of its high specific capacitance, chemical stability and thermal stability [20,21,22,23,24,25,26,27]. However, the main issue of RuO_2_ against its application as a pseudo-capacitive material lies in its particle aggregation, which may lead to compromised electrochemical performance [25,28,29]. The combination of RuO_2_ with carbon materials has been demonstrated to be an effective strategy to address this issue. For example, Shen et al. prepared a RuO_2_ nanodots/reduced graphene oxide composite as a pseudocapacitive material which demonstrated improved cycling stability due to the layered structure of the ultrathin carbon sheets [30]. Zhu et al. synthesized a carbon quantum dot-decorated RuO_2_ network. The composites exhibited a specific capacitance of 460 F g^−1^ and excellent rate capability [25]. 

Ti_3_C_2_, as a novel kind of 2D material, was prepared by selectively etching the Al element from Ti_3_AlC_2_ in hydrofluoric acid. Ti_3_C_2_ showed a promising performance as an energy storage material due to its unique accordion-like layered structure, electrolyte wettability and good metallic conductivity [1,2,3,31,32,33]. For example, Kurra et al. fabricated a coplanar micro-supercapacitor by using Ti_3_C_2_ as the electrode material [34]. Peng et al. also fabricated an all Ti_3_C_2_-based solid-state micro-supercapacitor [35]. These devices exhibited excellent capacitances and great cyclic performances. Moreover, Ti_3_C_2_ is regarded as an excellent host for metal oxide to construct high-performance active materials [36]. For instance, Xiong et al. designed a sandwich-like SnO_2_/Ti_3_C_2_/SnO_2_ architecture through a wet chemistry approach, and the composite delivered high reversible capacity and long-term cycling (up to 810 mAh g^−1^ after 200 cycles) when used as a lithium battery electrode material [36]. Rakhi et al. deposited MnO_2_ over Ti_3_C_2_ nanosheets, and the fabricated composite exhibited excellent cycling stability in the application of a supercapacitor [37]. Therefore, combining the advantages of metal oxide and Ti_3_C_2_ to prepare Ti_3_C_2_/metal oxide composites is an effective method to construct high-performance energy storage materials. 

Recently, Zhai and co-workers found that phosphate doping into Co_3_O_4_ could improve the fast electrode kinetics and stimulate high chemical reactivity [38]. Following a similar strategy, a phosphate ion-modified RuO_2_/ Ti_3_C_2_ composite (denoted as PRT) was fabricated in this work. As far as we know, phosphate ion-modified Ti_3_C_2_/RuO_2_ composites have not been reported as a supercapacitor electrode material. In general, the combination of phosphate ion, Ti_3_C_2_ and RuO_2_ can bring the following merits: (1) Ti_3_C_2_ serves as a support to prevent RuO_2_ particles from aggregating for longer cycling life; (2) the layered structure and high conductivity of Ti_3_C_2_ enables fast ion diffusion and fast electrons transfer, leading to a superior rate capability; (3) phosphate ion doping into RuO_2_ could highly improve the chemical reactivity of RuO_2_, leading to an enhancement in specific capacitance.

## 2. Materials and Methods

### 2.1. Material Preparation

All chemicals were used as received without further purification. Ti_3_C_2_ (11 technology Co., Ltd., Changchun, China). RuCl_3_·3H_2_O, NaOH, polytetrafluoroethylene (PTFE, 60%), H_2_SO_4_, NaH_2_PO_2_•H_2_O and other chemical reagents used in experiments were purchased from Adamas.

### 2.2. Synthesis of Phosphate Ion-Modified RuO_2_/Ti_3_C_2_

In a typical preparation process, 50 mg of Ti_3_C_2_ was added into an ethanol/water mixture with a ratio of 1:1 (*v:v*), followed by a sonication process for 1 h. Then, RuCl_3_·xH_2_O solution was dropped into the Ti_3_C_2_ suspension and stirred for 1 h. After that, the pH of the suspension was adjusted to 7 by 0.5 M NaOH solution. After being sonicated for 6 h, the RuO_2_/Ti_3_C_2_ composite was collected by centrifugation at 8000 rpm, rinsed with deionized waterand ethanol, then dried at 60 °C overnight. To obtain PRT, RuO_2_/Ti_3_C_2_ composite and NaH_2_PO_2_•H_2_O powder were placed in a combustion boat and annealed in a tube furnace filled with an Ar atmosphere at 250 °C for 1 h. 

In order to obtain optimal samples, three kinds of PRT were prepared by using 30 mg, 60 mg and 90 mg RuCl_3_·xH_2_O during the synthesis process (denoted as PRT-30, PRT-60 and PRT-90, respectively). The PRT sample in Figure 5 refers to PRT-60. 

### 2.3. Electrochemical Characterization

The working electrodes were prepared by pressing the mixture of 80 wt % as-prepared PRT materials, 10 wt % carbon black and 10 wt % PTFE binder onto stainless steel mesh. The mixture was then dried in a vacuum oven at 80 °C over 12 h. The pressure applied on the electrodes was 9 MPa. The mass loading of active material in every electrode was ~6 mg/cm^2^.Electrochemical measurements were carried out with a GAMRY Reference 3000 electrochemistry workstation in 1 M H_2_SO_4_ aqueous solution using a three-electrode mode. A platinum electrode was used as the counter electrode and an Ag/AgCl electrode was used as the reference electrode.

### 2.4. Material Characterization 

The crystal structures of the composites were characterized by an X-ray diffractometer (XRD; D/Max 2500 V PC, Cu-Ka radiation, Rigaku, Osaka, Japan) and X-ray photoelectron spectroscopy (XPS; Escalab250, Thermo Fisher Scientific Inc., MA, USA). The Brunauer–Emmett–Teller (BET) surface area of the samples was characterized by using the Micromeritics surface area and porosimetry system (ASAP 2420, Atlanta, GA, USA ). The morphology of the products was characterized by scanning electron microscopy (SEM, HITACHI S-3400N, 5 Kv, Hitachi Ltd., Tokyo, Japan) and transmission electron microscopy (TEM, JEM-2100, 200 kV, JEOL Ltd., Osaka, Japan). 

## 3. Results and Discussion

**Figure 1:**
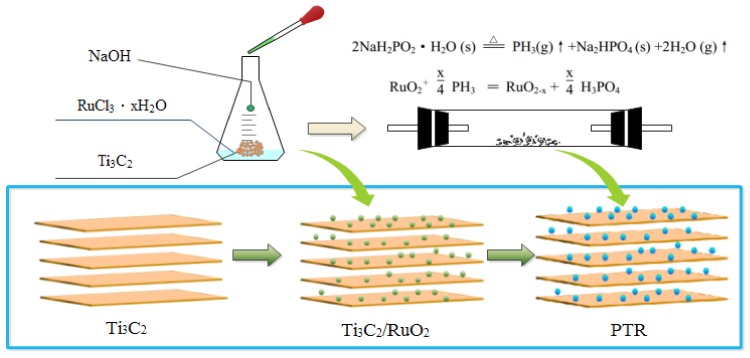
Schematic illustration of the procedure for preparing the phosphate ion-modified RuO_2_/ Ti_3_C_2_ composite (PRT).

The synthesis process of PRT is shown in Figure 1. The phosphate ion functionalization mechanism can be described as follows: Na_2_PO_2_•H_2_O was decomposed into PH_3_ gas, H_2_O gas and Na_2_HPO_4_ at high temperatures. After that, RuO_2_ was reduced to RuO_2−x_ by PH_3_ gas and H_2_PO_4_^−^ was introduced onto the surface of RuO_2−x_ at the same time. Finally, the phosphate ion-modified RuO_2_/Ti_3_C_2_ was collected. 

**Figure 2:**
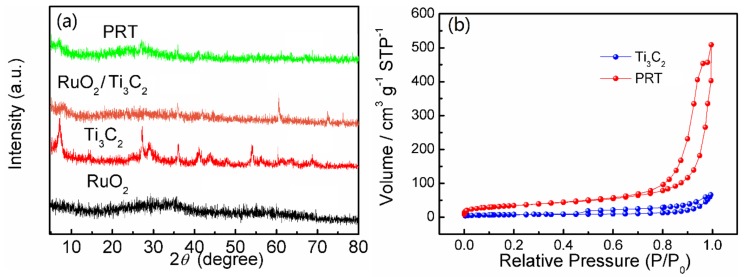
(**a**) X-ray diffractometry (XRD) patterns of RuO_2_, Ti_3_C_2_, RuO_2_/Ti_3_C_2_ and PRT; (**b**) BET nitrogen adsorption–desorption isotherms.

The structure and morphology of the products were investigated by XRD. The XRD curves (Figure 2a) of RuO_2_ showed no discernible peaks, indicating the amorphous state of RuO_2_. For Ti_3_C_2_, a prominent peak appeared at 2*ϴ* = 6.90°, characteristic of the (002) plane of Ti_3_C_2_, which fitted well with other related results [39,40,41]. For PRT, the peaks of Ti_3_C_2_ became weaker compared with the pure Ti_3_C_2_, which was mainly influenced by the introduction of RuO_2_ on the surface of the Ti_3_C_2_. Compared with RuO_2_/Ti_3_C_2_, PRT showed no discernible change, indicating that no phase transformation occurred during the annealing process. The nitrogen adsorption and desorption isotherms of Ti_3_C_2_ and PRT are shown in Figure 2b. The BET surface area value of the PRT sample was 120.33 m^2^ g^−1^, while Ti_3_C_2_ only possessed a surface area of 23.64 m^2^ g^−1^. The increased surface area of PRT suggests that the introduction of RuO_2_ on the surface of the Ti_3_C_2_ can effectively inhibit the restacking effect of Ti_3_C_2_ and prevent RuO_2_ particles from aggregating. 

**Figure 3:**
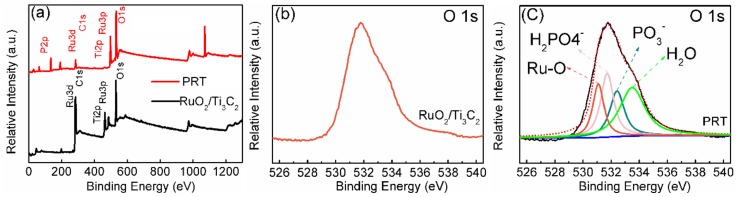
(**a**) X-ray photoelectron spectroscopy (XPS) survey of RuO_2_/Ti_3_C_2_ and PRT. High-resolution O1s XPS spectrum of (**b**) RuO_2_/Ti_3_C_2_ and (**c**) PRT.

XPS investigations were employed to survey the surface chemical composition and chemical bonding states of the products. Figure 3a shows the XPS survey of RuO_2_/Ti_3_C_2_ and PRT. Compared with RuO_2_/Ti_3_C_2_, the appearance of the P peak for PRT samples confirmed the phosphate doping. The O1s spectra at 531 eV shown in Figure 3b,c demonstrated the existence of RuO_2_ (the value from the literature is also 531 eV) [20]. The new characteristic peaks at 531.7 and 532.4 eV for PRT were related to the oxygen species of H_2_PO_4_^−^ and PO_3_^−^, respectively (values from the literature are 531.6 and 532.6 eV) [38,42]. The above results verify that the phosphate ions were successfully introduced on the surface of RuO_2_.

**Figure 4:**
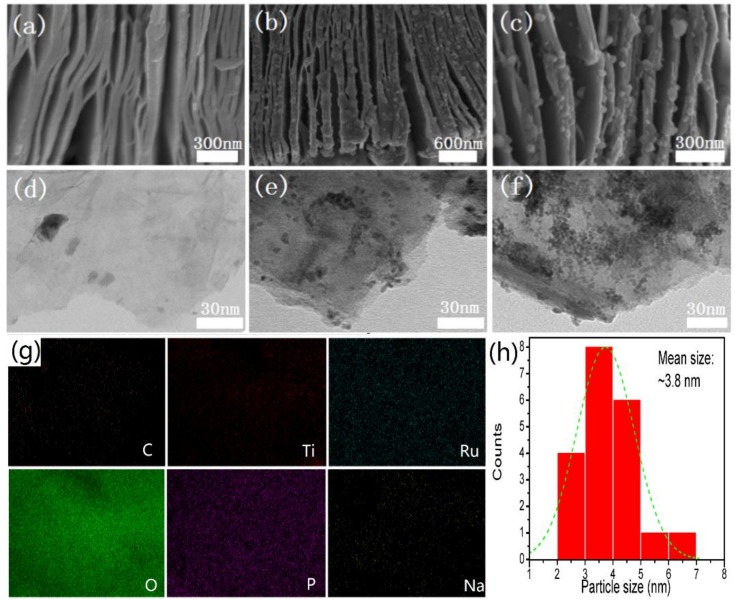
(**a**) SEM image of Ti_3_C_2_; (**b**) SEM image of RuO_2_/Ti_3_C_2_; (**c**) SEM image of PRT; (**d**) TEM image of Ti_3_C_2_; (**e**) TEM image of RuO_2_/Ti_3_C_2_; (**f**) TEM image of PRT; (**g**) element mapping of PRT; (**h**) size histogram of RuO_2_.

An SEM analysis was conducted to investigate the morphology of Ti_3_C_2_, RuO_2_/Ti_3_C_2_ and PRT. An SEM image of Ti_3_C_2_ is shown in Figure 4a. The impressive accordion-like layered structure of Ti_3_C_2_ can be observed. The interspaces between layers provide channels for material transfer and ion diffusion [36,37,43]. Figure 4b,c show the SEM images of RuO_2_/Ti_3_C_2_ and PRT, respectively. Obviously, the unique layered architecture was still maintained even after being subjected to a high-temperature process. At the same time, RuO_2_ particles were deposited on the surface of Ti_3_C_2_. Furthermore, PRT (Figure 4c) showed no morphology change compared with RuO_2_/Ti_3_C_2_ (Figure 4b), which is consistent with the XRD results. The layered architecture of PRT is expected to promote the reversible redox reactions and ion adsorption–desorption process.

The structures of Ti_3_C_2_, RuO_2_/Ti_3_C_2_ and PRT were also revealed by the TEM images. A typical TEM image of Ti_3_C_2_ sheets can be observed in Figure 4d. The transparent nanosheets of Ti_3_C_2_ mainly originated from the ultrasonic processing before the TEM was conducted. The TEM images of RuO_2_/Ti_3_C_2_ and PRT are shown in Figure 4e,f. Compared with Ti_3_C_2_ sheets (Figure 4d), nanoparticles were observed on the surface of Ti_3_C_2_. The Energy Dispersive Spectrometer (EDS) results (shown at the bottom of Figure 4g) further demonstrated the uniform distribution of RuO_2_ particles and phosphate doping. The dimensions of the RuO_2_ particles were calculated to be ~3.8 nm according to the TEM results (shown in Figure 4h). The appearance of the sodium element mainly comes from the residue of NaH_2_PO_2_•H_2_O. It has been reported that the phosphate ion functionalization strategy is an effective way to promote the performance of metal oxides [38,44]. After the phosphate ions were introduced onto the surface of metal oxides, longer bonds and smaller electronegativity can promote the surface reactivity and electrode kinetics of the metal oxides [45]. According to this theory, the phosphate ion-modified RuO_2_/Ti_3_C_2_ composite is expected to exhibit a better electrochemical performance than the pure RuO_2_ and RuO_2_/Ti_3_C_2_ samples when they are applied as supercapacitor electrode materials.

**Figure 5:**
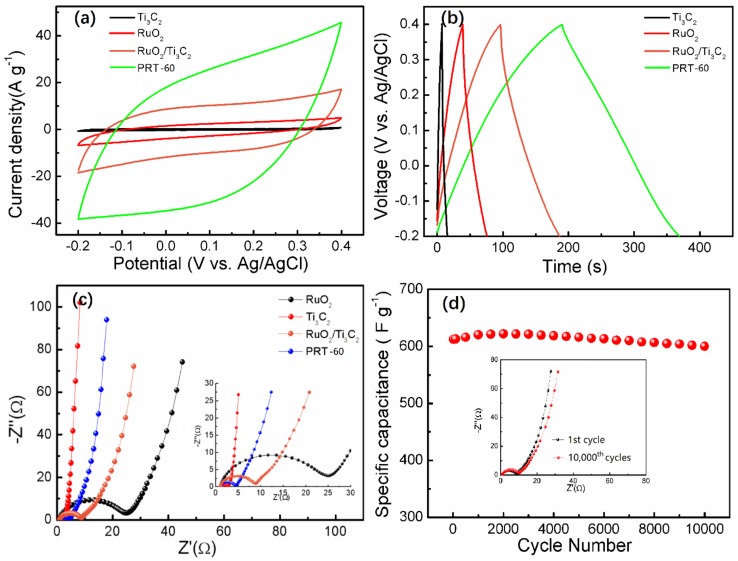
(**a**) Cyclic voltammetry (CV) curves, (**b**) galvanostatic charge–discharge (GCD) curves and (**c**) Nyquist plots of the Ti_3_C_2_, RuO_2_, RuO_2_/Ti_3_C_2_ and PRT-60 electrodes; (**d**) long-term cycling test by measuring 10,000 cycles at a constant current density of 2 A g^−1^ (the insert shows the EIS values cycles 1 and 10,000).

In order to verify the above inference, the electrochemical performances of Ti_3_C_2_, RuO_2_, RuO_2_/Ti_3_C_2_ and PRT-60 electrodes were first evaluated using a three-electrode system at the scan rate of 100 mV s^−1^ with 1 M H_2_SO_4_ as the solution. Figure 5a shows the cyclic voltammetry (CV) curves of these electrodes in a voltage window of −0.2 to 0.4 V (vs. Ag/AgCl). At any given scanning rate, the different integral areas of the CV curve indicated the difference in capacity [46,47]. Compared with Ti_3_C_2_, RuO_2_ and RuO_2_/Ti_3_C_2_ samples, the CV curve of PRT-60 showed the largest areas, indicating that it had the highest specific capacitance. Furthermore, even at the high scan rate of 100 mV s^−1^, the CV curve of PRT-60 still retained a relatively rectangular shape, implying the excellent rate capability and low contact resistance of the PRT-60 electrode. To investigate the electrochemical performances of the PRT-60 electrode, galvanostatic charge–discharge (GCD) cycling experiments were conducted at a current density of 2 A g^−1^. As shown in Figure 5b, the symmetrical triangle shape of the GCD curves with a small internal resistance (IR)-drop demonstrated the excellent reversibility and charge–discharge properties. The electrode’s specific capacity was calculated according to the equation: $C_{S}$ = IΔt/mΔV, where $C_{S}$ (F g^−1^) is the mass specific capacitance, *I* (A) is the discharge current, Δt (s) is the discharge time, *m* (g) is mass loading of active component and ΔV (V) is potential window [48]. The specific capacitances of Ti_3_C_2_, RuO_2_, RuO_2_/Ti_3_C_2_ and PRT-60 electrodes were calculated to be 24.97 F g^−1^, 125.7 F g^−1^, 306.6 F g^−1^ and 612.7 F g^−1^, respectively. The PRT-60 electrode exhibited the maximum specific capacitance, which is in accordance with the CV results mentioned above. This improvement in electrochemical properties for the PRT-60 electrode can be attributed to the synergistic effects of phosphate ion modification and the introduction of Ti_3_C_2_. 

To further investigate the advantages of these composite materials, an electrical impedance spectroscopy (EIS) measurement was conducted. The Nyquist impedance plots of the EIS results are shown in Figure 5c. As we know, the diameter of the semicircular curve at a high frequency represents the charge transfer resistance (*R_ct_*) at the electrode–electrolyte interface [49]. In particular, the RuO_2_/Ti_3_C_2_ electrode exhibited a lower *R_ct_* than that of pure RuO_2_, indicating that the layered Ti_3_C_2_ is beneficial for fast electrode kinetics. What is more, PRT-60 exhibited an even lower *R_ct_* than RuO_2_/Ti_3_C_2_, illustrating that phosphate ion modification helps to accelerate the charge transfer process.

The long-term cycle performance of electrodes is a crucial parameter in practical application. Therefore, the cycle stability of PRT-60 was evaluated by repeating the GCD test for 10,000 cycles at a current density of 2 A g^−1^. As shown in Figure 5d, the increase of the capacitance at the beginning cycles may be caused by the presence of the activation process in the electrode, which is commonly observed for metal oxides [49,50]. After 10,000 cycles, the specific capacitance was still maintained at 600.14 F g^−1^, and only decreased by 2.05% of the initial capacitance (612.72 F g^−1^). 

The EIS analysis of PRT-60 was measured at the cycles 1 and 10,000. As shown in the insert of Figure 4d, the EIS values were almost similar, further demonstrating the electrochemical stability. In view of the above discussions, we conclude that the excellent performance of PRT-60 can be ascribed to the following reasons: First, the existence of Ti_3_C_2_ not only improves the electrical conductivity of the composite but also provides channel that facilitates the ion diffusion of the electrolyte. Furthermore, the phosphate ion-functionalized RuO_2_ provides a higher number of active reaction sites and accelerates the charge transfer process, resulting in an excellent electrochemical performance.

**Figure 6:**
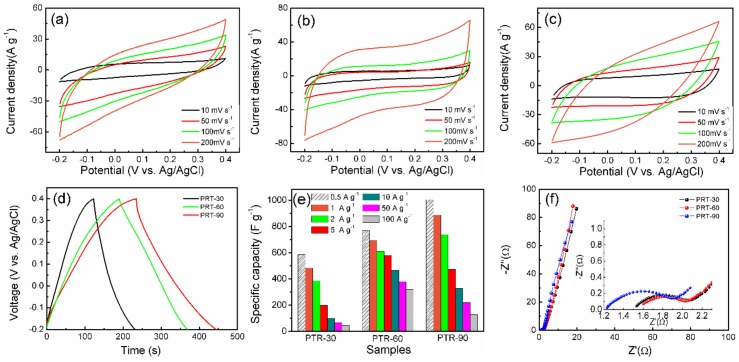
CV curves of (**a**) PRT-30, (**b**) PRT-60 and (**c**) PRT-90 electrodes at scan rates varying from 10 to 200 mV s^−1^; (**d**) GCD curves of the three kinds of samples; (**e**) histogram of the specific capacity for the three kinds of samples at different current densities; (**f**) Nyquist plots of the samples (the inset is the expanded view of the high-frequency range).

In order to obtain the optimized samples, three kinds of PRT with various RuO_2_ loadings were prepared by using 30 mg, 60 mg and 90 mg RuCl_3_·xH_2_O during the synthesis process (denoted as PRT-30, PRT-60 and PRT-90, respectively). CV curves with different scan rates are shown in Figure 6a–c. Obviously, the CV curves of all samples retained a typical rectangular shape at a low sweep rate, indicating a good charge transfer process [51]. However, at a high scanning rate of 200 mV s^−1^, the curves of PRT-30 and PRT-90 electrodes deviated from the rectangular shape, while PRT-60 still retained the ideal shape. This suggests that reasonable mass loading is beneficial for rate capability. This advantage also applies to the electrochemical capacity. As shown in Figure 6e and Table 1, the excellent rate capability of PRT-60 is also reflected by the histogram of the specific capacity at different current densities. When the current density was less than 2 A g^−1^, PRT-90 exhibited the highest capacity compared with the other two samples. However, with the increase of the current density, the capacity of all three samples showed a declining trend and the capacity of PRT-90 (328.67 F g^−1^) was smaller than that of PRT-60 (466.87 F g^−1^) when the current density reached 10 A g^−1^. Even at a high current density of 100 A g^−1^, the PRT-60 electrode still delivered a high capacitance of 320.83 F g^−1^, which was about 2.5 times larger than that of PRT-90 (128.65 F g^−1^) electrode and seven times larger than that of PRT-30 (45.85 F g^−1^). This result is in accordance with the CV results shown in Figure 6a–c. The obtained PRT-60 also showed better performance compared with other reported RuO_2_-based materials (as shown in Table 2). Figure 6f compares the Nyquist plots of the three samples. The electronic resistance (*R_s_*) of these samples increased with the increase of ruthenium oxide content, further confirming the effect of Ti_3_C_2_ on enhancing electronic conductivity. When the proportion of Ti_3_C_2_ nanosheets decreased, the conductivity of the composites began to decrease as well.

According to the above analysis of electrochemical properties, it is evident that PRT-60 delivered better electrochemical performance than the other two samples. (I) For PRT-30, the low content of RuO_2_ led to a lower capacity compared with the other two samples. (II) For PRT-90, while it showed the highest discharge capacity at a low current density, its capacity attenuation was the most serious at a high current density (only 128.65 F g^−1^ at 100 A g^−1^). The poor rate capacity of PRT-90 is mainly due to the accumulation of excessive RuO_2_ nanoparticles, which occupy the spaces between the Ti_3_C_2_ layers and prevent the electrolyte from entering.

## 4. Conclusions

In summary, a phosphate ion-modified RuO_2_/Ti_3_C_2_ composite was successfully synthesized and applied in a supercapacitor electrode. The resulting PRT composite combines the advantages of Ti_3_C_2_, RuO_2_ and phosphate ion modification. Morphological and chemical characterization indicated that the Ti_3_C_2_ not only acts as a conductive substrate to prevent RuO_2_ nanoparticles from aggregating, but also provides electrolyte-filled channels for ion diffusion. Electrochemical studies showed that the phosphate ion modification can greatly improve the electrochemical properties of the composites. The PRT composite exhibited a specific capacitance of 612.7 F g^−1^ at 2.0 A g^−1^ and a satisfactory rate capacity (320.83 F g^−1^ at 100 A g^−1^). In addition, the capacitance of PRT could retain 97.95% (600.14 F g^−1^) of the original value after 10,000 cycles at a current density of 2 A g^−1^. More importantly, the method delivered in our experiment can be also applied to other energy storage materials.

## Figures and Tables

**Table 1:** Specific capacitance of PRT-30, PRT-60 and PRT-90 at different current densities.

Samples	Specific Capacitance (F g^−1^)						
	0.5 A g^−1^	1 A g^−1^	2 A g^−1^	5 A g^−1^	10 A g^−1^	50 A g^−1^	100 A g^−1^
PRT-30	585.04	484.63	384.62	199.91	100.25	66.92	45.85
PRT-60	768.03	693.02	612.72	578.02	466.87	380.64	320.83
PRT-90	1004.3	888.54	737.59	474.42	328.67	220.52	128.65

**Table 2:** Comparison of PRT with other reported RuO_2_ capacitors.

Material	Capacitance (F g^−1^)	Cycle Life (Cycles)	Reference
Reduced graphene oxide sheets modified with RuO_2_	400	2500	[9]
RuO_2_/reduced graphene oxide nanocomposites	489	1000	[19]
RuO_2_ deposited on the surface of graphene sheets	551	2000	[27]
Reduced graphene oxide–RuO_2_ hybrid materials	509	2000	[29]
Phosphate ion-modified RuO_2_/Ti_3_C_2_ composite	693	10,000	This work
